# Mapping the evidence on oral behaviors and mental health: a scoping review with implications for integrated oral healthcare

**DOI:** 10.3389/froh.2026.1906938

**Published:** 2026-10-01

**Authors:** Diego De Nordenflycht, Gabriela Curtze, Camila Antúnez, Rosario Espinoza, Javier Morán-Kneer, Giancarlo De La Torre Canales

**Affiliations:** 1 Faculty of Dentistry, Universidad Andres Bello, Viña del Mar, Chile; 2 Private Practice, Viña del Mar, Chile; 3 Center for Translational Studies in Stress and Mental Health (C-ESTRES), Universidad de Valparaíso, Valparaíso, Chile; 4 Egas Moniz Center for Interdisciplinary Research (CiiEM); Egas Moniz School of Health & Science, Caparica, Almada, Portugal; 5 Department of Dental Medicine, Karolinska Institutet, Huddinge, Sweden

**Keywords:** mental health, oral behaviors, oral parafunctions, psychological factors, stress

## Abstract

**Objective:**

To map and characterize the available evidence on the associations between oral behaviors and psychological factors by identifying the psychological constructs investigated and describing the range and patterns of these associations across different oral behaviors.

**Method:**

Electronic searches were conducted in PubMed, Web of Science and Scopus (November 2024; updated August 2025), using controlled vocabulary and terms related to oral behaviors and psychological factors. Studies published in the last 10 years assessing oral behaviors and psychological factors were eligible. Study selection followed the PRISMA-ScR guidelines, with two independent reviewers screening titles, abstracts, and full-texts. Full-text articles were obtained from the records after applying the inclusion/exclusion criteria, and findings were synthesized narratively, focusing on methodological characteristics, diagnostic assessment, and reported associations.

**Results:**

Forty-two studies met the eligibility criteria. Most were cross-sectional and showed substantial heterogeneity in terminology, assessment methods, and psychological constructs examined. While five studies reported no significant associations, the majority identified associations between oral behaviors and multiple analytical domains of psychological factors. The analytical domains “anxiety–obsessive/compulsive” and “mood/affective” were the most frequently represented among reported associations, particularly in studies of awake bruxism and nail-biting. Additional associations were reported with somatic symptom disorders, personality traits, emotional processing, and cognitive–executive functioning.

**Conclusion:**

Current evidence suggests that oral behaviors are associated with psychological factors spanning multiple analytical domains, particularly “anxiety and obsessive–compulsive symptoms” and “mood and affective symptoms”. However, the evidence base remains predominantly cross-sectional and methodologically heterogeneous, limiting causal inference, comparisons across studies, and the certainty with which these associations can be interpreted.

## 1 Introduction

Body-focused repetitive behaviors (BFRBs) is an umbrella term for undesirable, repetitive motor activities that may result in functional impairment (1). Although many individuals engage in harmless “nervous habits”, the term BFRBs refers specifically to behaviors that lie at the maladaptive end of the behavioral continuum and are associated with clinically relevant impairment or distress (2). These non-functional behaviors include, but are not limited to, hair-pulling (trichotillomania), skin-picking (dermatillomania), finger cracking, and several oral behaviors such as nail-biting (onychophagia), cheek chewing, lip biting, finger sucking, and teeth grinding (1, 2). These oral behaviors, often referred to as “parafunctional oral behaviors”, comprise a group of activities that differ from those required for, or associated with, normal jaw functions such as mastication, swallowing, communication, or breathing (3). According to the American Academy of Pediatric Dentistry, harmful oral behaviors include finger sucking, pacifier sucking, lip sucking and biting, self-injurious habits, mouth breathing, and tongue thrusting (4). Some form of repetitive oral behavior affects up to 90% of the population (5, 6), but prevalence estimates vary widely across studies and appear to be higher in males, depending on factors such as age, race, country, sex, dietary habits, socioeconomic status, maternal education, and occupation (7).

Parafunctional oral behaviors may lead to oral or non-oral consequences, with their impact depending on the nature, onset and duration of the behavior (8). Regarding oral consequences, associations have been reported between non-nutritive sucking behavior i.e., sucking of fingers, pacifiers, or other objects, and malocclusions such as Class II canine relationship, posterior crossbite, and anterior open bite (9–12). In addition, nail-biting has been associated with gingival swelling, gingival abscesses, infections due to foreign body embedment, apical root resorption, dental malocclusion, as well as crowding and rotation of the incisors (13).

With respect to non-oral consequences, subjects reporting multiple parafunctional oral behaviors have been shown to present a 16-fold increase in the odds of chronic temporomandibular disorders (TMDs) compared with those reporting only a few behaviors (14). Furthermore, a recent systematic review demonstrated an association between bottle and pacifier use and asthma in children (15). Nail-biting, probably the most extensively studied parafunctional oral behavior, has been associated with damage to the nail matrix, nail bed, nail plate, and periungual skin, leading to progressive nail shortening and loss of the distal nail bed (13, 16).

According to the most recent edition of the Diagnostic and statistical manual of mental disorders (DSM-5-TR), BFRBs such as hair-pulling and skin-picking are classified within the group of obsessive-compulsive and related disorders. Other behaviors, including parafunctional oral behaviors (i.e., nail-biting, lip/cheek biting, tongue chewing) are referred as examples of BFRBs, and are typically coded under “Other Specified Obsessive-Compulsive and Related Disorder” (17). The etiopathogenesis of BFRBs remains incompletely understood, as existing findings are often inconsistent or controversial. Current behavioral models consistently emphasize the importance of psychological factors in the development and maintenance of BFRBs (1). Contemporary empirical research conceptualizes psychological factors as internal dimensions of psychological functioning, encompassing enduring personality traits (stable patterns of experiencing and interpreting self and others), psychological symptoms or distress states (e.g., anxiety, tension, urges), and regulatory-cognitive-affective functions (e.g., emotion regulation, inhibitory control, mentalization). Although these factors influence behaviors, they are not directly observable without self-report or psychological assessment (18, 19).

For the purpose of the present review, the term “oral behaviors” is used as an umbrella construct to encompass a heterogeneous group of repetitive or sustained oral motor activities not directly related to functional tasks such as mastication, swallowing, or breathing. This includes behaviors traditionally classified as BFRBs (e.g., nail-biting, lip/cheek biting), as well as other oral activities commonly described in dental literature as parafunctional habits (e.g., awake bruxism, object biting, sucking behaviors). Given the conceptual overlap but lack of unified terminology across disciplines, this broader definition was adopted to allow an inclusive mapping of the available evidence.

Despite a growing number of studies exploring oral behaviors in relation to psychological factors (20), the available evidence remains fragmented across dental, medical, and psychological research. Variability in terminology, assessment tools, and theoretical frameworks has limited a comprehensive understanding of how these behaviors relate to mental health (7). Mapping this evidence is therefore necessary to clarify the nature and extent of these associations and to identify methodological and conceptual gaps that hinder the development of diagnostic and theoretical consensus. Accordingly, this scoping review aims to map and characterize the available evidence on associations between oral behaviors and psychological factors by identifying the psychological constructs investigated and describing the range and patterns of these associations across different oral behaviors. By integrating evidence from dental, medical, and psychological research, this review seeks to provide a conceptual bridge between disciplines and to clarify the role of oral behaviors within a behavioral and mental health framework, which may contribute to advancing the understanding of oral behaviors beyond the dental perspective and support their recognition as clinically relevant indicators of psychological processes.

## 2 Method

### 2.1 Protocol

This scoping review was conducted in accordance with the Preferred Reporting Items for Systematic Reviews and Meta-Analyses extension for Scoping Reviews (PRISMA-ScR) (21). The research question for this scoping review was “what psychological factors have been associated with oral behaviors and how these associations have been conceptualized and assessed?”.

### 2.2 Eligibility criteria

The present review included analytical observational studies (cross-sectional, case-control, prospective cohort, retrospective cohort, and other observational designs) published within the last 10 years, written in English, and conducted in children, adolescents or adults of both sexes. Eligible studies assessed oral behaviors using questionnaires and/or standardized clinical examinations and evaluated psychological factors through psychometric instruments, diagnostic assessments, investigator-developed questionnaires, or other self-reported measures, reporting statistical analyses of associations between these variables. Oral behaviors were operationally defined according to the conceptual framework described in the Introduction, including both body-focused repetitive behaviors involving the oral region and parafunctional oral activities assessed through self-report or clinical examination. Studies involving participants with diagnosed psychiatric disorders were considered eligible provided they met the predefined inclusion criteria and reported extractable data on oral behaviors and psychological factors. When studies included mixed populations or assessed multiple body-focused repetitive behaviors, they were included only if data specifically related to oral behaviors could be clearly identified and extracted.

In studies where psychological factors were assessed alongside psychosocial or contextual factors, only data related to psychological factors were considered. Studies reporting body-focused repetitive behaviors (BFRBs) involving oral behaviors were included only when data on oral behaviors and their associations could be clearly isolated for extraction. Studies in which sleep bruxism was considered as an oral behavior were excluded, unless wake-time oral behaviors were also assessed and reported separately. Studies focusing on prolonged bottle feeding or breastfeeding were excluded due to the difficulties in distinguishing physiological behaviors from parafunctional behaviors. Narrative and systematic reviews, and non-human based studies (such as technical notes, animal-based studies) were also excluded.

### 2.3 Information sources and search strategy

A systematic search of the medical literature was carried out in PubMed, Web of Science and Scopus in November 2024 and subsequently updated in August 2025. The search strategy combined controlled vocabulary (e.g., MeSH terms in PubMed) and free-text terms. Synonymous terms within each conceptual domain were combined using the Boolean operator OR, whereas the oral behavior and psychological factor domains were combined using AND. The search strategy combined controlled vocabulary (e.g., MeSH terms in PubMed) and free-text terms, covering two main conceptual domains: (1) oral behaviors (including awake bruxism, nail biting, lip and cheek biting, object biting, sucking habits, and oral parafunctions) and (2) psychological factors (including anxiety, depression, stress, obsessive-compulsive symptoms, personality traits, emotion regulation, and related constructs). Search terms were adapted to the indexing system of each database. Search strings were adapted to the indexing system and syntax requirements of each database. The complete search syntax for each database is provided in Supplementary Material 1. Only published articles addressing the research objective were considered.

### 2.4 Selection of sources of evidence

Two reviewers (GC and RE) independently screened the titles and abstracts of all records identified through the search strategy. Full-text articles were obtained for records meeting the inclusion/exclusion criteria. Inter-reviewer agreement during title and abstract screening was 90.9%, with a Cohen's *κ* of 0.46. For articles for which full access was not available, the corresponding author was contacted at least three times. Disagreements regarding study inclusion were first discussed between the two reviewers and, when consensus could not be reached, were resolved by a third reviewer (DDeN). Reference lists from included full-text articles were also cross-checked to identify additional relevant studies not captured by the electronic search.

### 2.5 Data charting process

A structured data-charting spreadsheet was developed by three reviewers (GC, RE and CA) to guide data extraction. Three reviewers independently charted the data and discussed the extracted information to ensure consistency and completeness. Any discrepancies were resolved by a fourth reviewer (DDeN). To facilitate evidence mapping, the individual psychological constructs extracted from the included studies were grouped into six analytical domains according to their conceptual similarity. This organizational scheme was developed specifically for the present review to synthesize the heterogeneous psychological variables reported across studies and does not represent an established psychological taxonomy. Operational definitions and categorization of the principal data items are provided in Supplementary Material 3. Oral behaviors were defined as repetitive or non-nutritive oral activities explicitly assessed in the included studies. Individual behaviors were categorized according to the operational definitions used in the original studies and grouped under common labels only when the reported behaviors were clearly equivalent; no uniform diagnostic definition was retrospectively imposed. Psychological factors comprised the psychological, affective, cognitive, personality-related, somatic, and regulatory constructs assessed in relation to oral behaviors. The six analytical domains represent a review-specific organizational scheme based on conceptual similarity and do not constitute an established psychological taxonomy. Psychological assessment methods were descriptively classified as validated psychometric instruments, diagnostic assessments, investigator-developed questionnaires, or single-item/self-reported measures, according to the assessment approach reported in each study. These categories were used to characterize measurement heterogeneity and were not interpreted as a formal hierarchy of methodological quality.

### 2.6 Data items and synthesis of the results

For evidence mapping, an association was recorded when the original study reported a statistically significant relationship between an oral behavior and a psychological factor, including correlations, between-group differences, or regression-based associations. Associations were recorded according to the direction and statistical interpretation reported by the original study. The evidence was synthesized narratively and presented using summary tables and graphical representations. Results were organized across three main categories: (i) methodological characteristics (study design, No. of subjects included, included subjects phase of life, age range and age mean of included subjects, and terminology used to refer to “oral behaviors”), (ii) diagnostic aspects (oral behavior assessed, oral behavior assessment method, psychological factors, psychological factors assessment methods, and other non-oral body-focused repetitive behaviors assessed), and (iii) main outcomes (use of predictive multivariate model with oral behavior as main outcome, associations of oral behaviors with psychological factors). Because substantial heterogeneity was anticipated in terminology, psychological constructs, assessment methods, and study designs, findings were synthesized descriptively using an evidence-mapping approach rather than through quantitative comparison of associations across studies.

## 3 Results

### 3.1 Selection of sources of evidence

A total of 12,450 articles were identified through database searches. After removal of duplicates, 5,804 records were screened based on titles and abstracts. Of these, 150 articles were selected for full-text assessment, and 111 were excluded for various reasons. Three additional articles were identified through manual searching and included in the full-text evaluation. Hence, 42 studies were included in this scoping review. The study selection process is illustrated in the PRISMA flow diagram (Figure 1).

**Figure 1:**
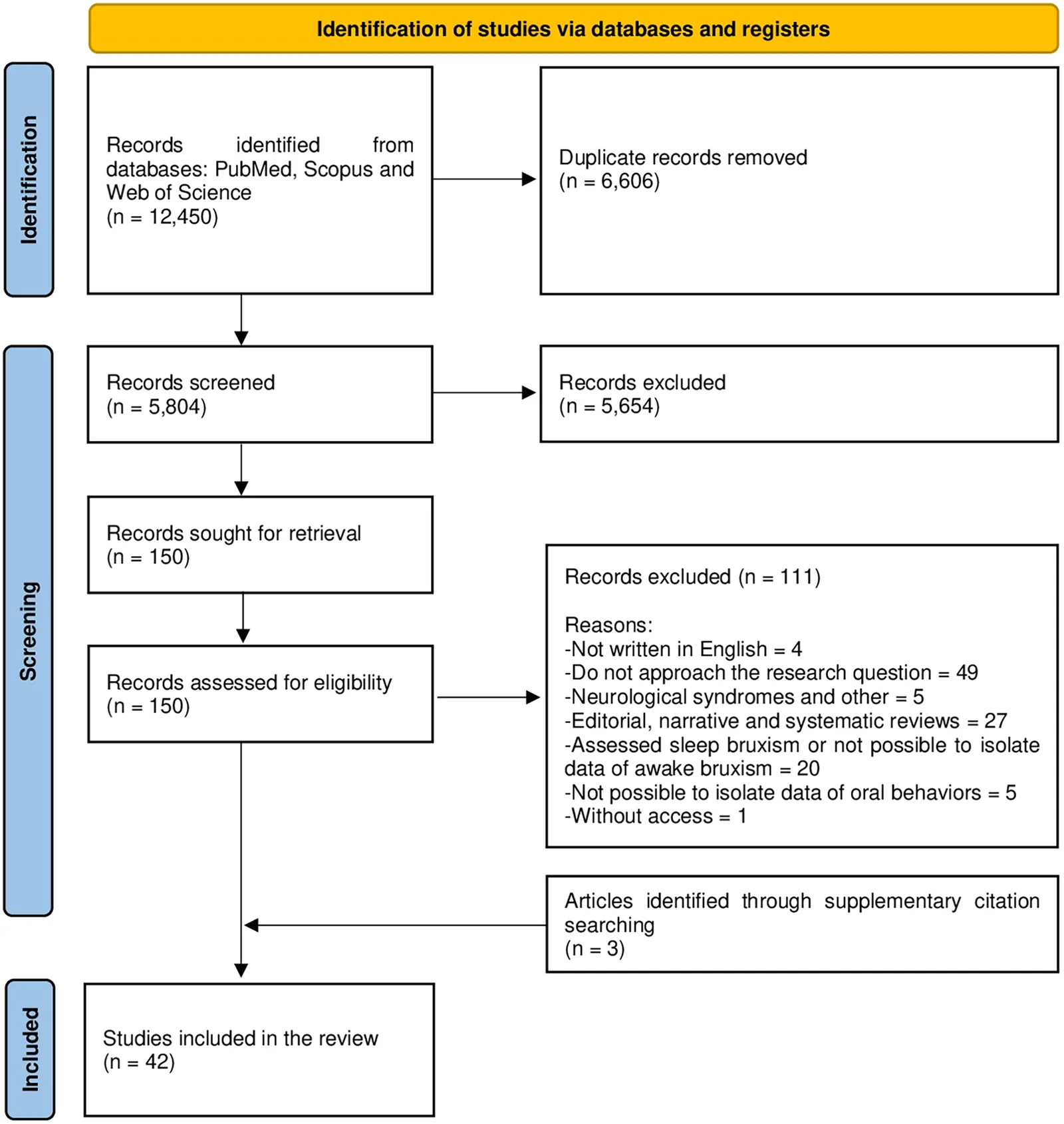
Flow chart of articles selection.

### 3.2 Characteristics of the sources of evidence

The included studies were published between 2014 and 2025 (22–63) originated from clinical and research centers located in Australia (34), Brazil (24, 42, 50, 51, 53, 54, 58, 60), Canada (41, 47), China (48), Colombia (62), Croatia (49), Egypt (52), El Salvador (28), Israel (25, 30, 37, 45), Italy (29, 55, 56, 63), Japan (35), Lithuania (39), The Netherlands (38, 57), Poland (22, 36), Romania (59), Saudi Arabia (26, 44), South Korea (61), Spain (43), Turkey (23, 32, 33, 46), and the United States (27, 31, 40).

### 3.3 Synthesis of results

The methodological characteristics, diagnostic characteristics, and main outcomes of the included studies are summarized in Supplementary Material 2.

#### 3.3.1 Methodological characteristics

The study designs of the included articles comprised one longitudinal observational study (38), one prospective cohort study (42), two retrospective cross-sectional studies (57, 63), four case-control studies (23, 31, 43, 58), and 34 cross-sectional studies (22, 24–30, 32–37, 39–41, 44–56, 59–62). Sample size ranged from 18 (50) to 5,328 participants (35), with a median of 263 subjects included. Regarding the populations studied, 31 studies included adults (22, 25–27, 29, 31, 34, 35, 38–51, 53, 55–61, 63), 14 included adolescents (23, 24, 26, 28, 30, 32, 33, 37, 42, 43, 46, 48, 54, 62), and five included children (23, 24, 28, 33, 52). Most studies recruited participants from school settings (24, 30, 32, 37, 46, 52, 54) or universities (22, 29, 34, 39, 41, 43, 45, 46, 53, 55, 56, 60). Several studies involve samples drawn from the general community (27, 28, 31, 35, 38, 44, 47, 50, 59), whereas others included clinical populations (23, 25, 33, 36, 42, 48, 49, 57, 58, 61–63). A wide range of terminology was used to describe “oral behaviors”. The most frequently associated terms were “body-focused repetitive behaviors” (28, 31, 32, 34, 35, 40, 46, 47), “oral parafunctional habits” (23, 44, 53), “oral parafunctional hyperactivity” (25), “oral behaviors” (41, 48, 61), “oral parafunctional behaviors” (27, 42, 49), “deleterious oral habits” (24, 33), and “oral habits” (52). More recent studies introduced more complex terms such as “non-functional waking-state oral behaviors” (57), “non-physiological awake oral behaviors” (62), and “non-functional oral behaviors” (63).

#### 3.3.2 Diagnostic aspects

The oral behaviors most frequently assessed were awake bruxism (25, 26, 29, 30, 36–39, 42, 43, 45, 50–56, 58–60, 62), nail-biting (22, 23, 28, 31, 32, 34, 35, 40, 44, 46, 47, 52), lip or cheek biting (23, 34, 40, 44), sucking habits (such as lip or finger sucking) (23, 52), objects biting (23, 44), and jaw play (i.e., performing small non-functional mandibular movements without tooth contact) (25). In addition, several studies evaluated a heterogeneous set of behaviors using non-specific terms such as “oral behaviors” or similar expressions (24, 25, 27, 33, 41, 48, 49, 57, 61–63).

Methods used to assess oral behaviors vary considerably across studies. The most common approaches were questionnaires based on dichotomous (yes/no) responses (23, 25, 26, 30, 32, 36, 37, 39, 43, 45, 60), Likert-type scales (29, 35), frequency-based scales (23, 44), or assessment methods that were not clearly specified (22, 31, 33, 46, 58, 59). Some studies employed standardized instruments, including the Oral Behavior Checklist (OBC) (27, 41, 42, 48–51, 53, 56, 57, 61–63), an adapted version of the Massachusetts General Hospital Hair-Pulling Scale (MGH-HPS) for oral behaviors (34, 47), the Nordic Orofacial Test-Screening (NOT-S) (24, 52), and a smartphone-based application for assessment of awake bruxism (55).

The psychological constructs extracted from the included studies were organized into six analytical domains according to their conceptual similarity: (1) “Anxiety and obsessive–compulsive symptoms” (including anxiety symptoms (22, 24, 25, 31–34, 36, 38, 40–44, 48–51, 53, 55–57, 59, 61–63), dysmorphic concern (34), fear of negative evaluation (34), obsessive-compulsive symptoms (22, 34), social phobia (25, 34), stress symptoms (26, 29, 30, 32, 34, 36–40, 43, 45, 46, 51, 57, 59, 60, 62), and post-traumatic stress disorder symptoms (58)); (2) “Mood and affective symptoms” (including anger (38), depression symptoms (24, 25, 27, 31–34, 40, 42, 43, 48, 49, 51, 55–59, 61, 62), lower psychological resilience (52), negative self-image (32), sadness (30, 54), self-criticism (47), and shame (47)); (3) “Somatic symptoms” (including attention to pain (42), somatic amplification (41, 49), somatic symptoms (27), and somatization (32, 43, 62)); (4) “Personality-related traits and symptoms” (including dependency (47), hostility (32), perfectionism (47), and personality traits (35, 43, 44)); (5) “emotional processing and regulation” (including alexithymia (36), cognitive emotion regulation strategies (47), emotional processing (36), emotion regulation difficulties (47), and stress coping (43)), and (6) “Cognitive, executive, and behavioral functioning” (including behavioral and emotional functioning (28), cognitive flexibility (31), hyperactivity (23), and response inhibition (31)). The organization of psychological constructs into six analytical domains assessed in the selected studies is summarized in Table 1.

**Table 1:** Classification of psychological factors assessed in the selected studies.

Domain	DSM-5-TR correspondence	Psychological factors included
Anxiety and obsessive–compulsive symptoms	Anxiety Disorders; Obsessive–Compulsive and Related Disorders	Anxiety, dysmorphic concern, fear of negative evaluation, obsessive–compulsive symptoms, perfectionism, social phobia, stress, symptoms of post-traumatic stress disorder symptoms
Mood and affective symptoms	Depressive Disorders	Anger, depression, lower psychological resilience, negative self, sadness, self-criticism, shame
Somatic symptoms	Somatic Symptom and Related Disorders	Attention to pain, somatic amplification, somatic symptoms, somatization.
Personality-related traits and symptoms	Personality Disorders	Dependency, hostility, perfectionism, personality traits.
Emotional Processing and Regulation	Psychological and behavioral factors affecting other medical conditions.	Alexithymia, cognitive emotion regulation strategies, emotional processing, emotion regulation difficulties, stress coping (coping strategies).
Cognitive, Executive, and Behavioral Functioning	Neurodevelopmental Disorders; Neurocognitive Functions	Behavioral and emotional functioning, cognitive flexibility, hyperactivity, response inhibition.

Similarly, assessment methods for psychological factors also showed substantial variability. The most frequently used instruments for anxiety symptoms assessment were the State- and Trait-Anxiety Inventory, (STAI) (33, 36, 38, 41, 43, 53), the General anxiety disorder-7 (GAD-7) (27, 34, 37, 48–50, 57, 61–63), and the Hamilton Anxiety Rating Scale (HAM-A) (31, 44). Stress symptoms were most commonly evaluated using the Perceived Stress Scale (PSS) (29, 36–39, 45, 46, 50, 60), while depressive symptoms were assessed using the Patient Health Questionnaire-9 (PHQ-9) (27, 34, 48, 49, 57, 61), the Children's Depression Inventory (CDI) (24, 33), and the Beck Depression Inventory (BDI) (25, 42). Less frequently used instruments are reported in Supplementary Material 2. Other non-oral BFRBs assessed in the selected studies were onychotillomania (22), hair-pulling, skin-picking (28, 34), and leg-shaking (35).

#### 3.3.3 Main outcomes

Several studies reported predictive multivariate models with oral behaviors as the primary outcome (24–27, 30, 35, 37–41, 44, 47, 49, 53, 54, 57–63). Five studies reported no statistically significant associations between oral behaviors and psychological factors (22, 31, 33, 34, 39). In contrast, other studies identified statistically significant associations between the same variables across different domains. Within the analytical domain of “Anxiety and obsessive–compulsive symptoms” associations were reported with awake bruxism, clenching, grinding, jaw play, lip or cheek biting, nail-biting, objects biting, “oral behaviors” (as a non-divisible ensemble of diverse behaviors), sucking habits, and toenail biting. “Mood and affective symptoms” were associated with awake bruxism, nail-biting, oral behaviors, and sucking habits. “Somatic symptoms” were associated with awake bruxism, nail-biting, and oral behaviors (non-divisible ensemble of behaviors). “Personality-related traits and symptoms” were associated with awake bruxism, clenching, and nail-biting. “Emotional processing and regulation” factors were associated with awake bruxism, and nail-biting, whereas “Cognitive, executive, and behavioral functioning” factors were associated with nail-biting, objects biting, and toenail biting. Detailed associations between specific oral behaviors and psychological factors are presented in Table 2 and graphically summarized in Figure 2. Additionally, three studies reported statistically significant associations involving both oral and non-oral BFRBs in relation to psychological factors such as anxiety, depression, obsessive-compulsive symptoms, body dysmorphic disorder, and fear of negative evaluation (34), child anxiety, anxiogenic parenting behaviors (40), maladaptive cognitive emotion regulation strategies and shame (47).

**Table 2:** Associations between oral behaviors and specific psychological factors. OBs: oral behaviors reported as a non-divisible ensemble of diverse behaviors.

Oral Behavior	Domain	Psychological factors
Awake bruxism	Domain	Anxiety symptoms (36–38, 42, 43, 45, 50, 51, 53, 55, 56, 62)
		Post-traumatic stress disorder symptoms (58)
		Social phobia (25)
		Stress symptoms (26, 29, 30, 36–38, 45, 51, 59, 60, 62)
	Domain	Depression symptoms (42, 45, 51, 55, 56, 62)
		Lower psychological resilience (52)
		Sadness (30, 54)
	Domain	Somatization (43, 45, 62)
	Domain	Personality: Neuroticism (43)
	Domain	Alexithymia (36)
		Stress coping: Adapted coping strategies (43)
Clenching	Domain	Anxiety symptoms (44)
	Domain	Personality: Extraversion (44)
Grinding	Domain	Anxiety symptoms (44)
Jaw play	Domain	Social phobia (25)
Lip or cheek biting	Domain	Anxiety symptoms (40, 44)
Nail-biting	Domain	Anxiety symptoms (32, 40, 44)
		Stress symptoms (46)
	Domain	Depression symptoms (32)
		Lower psychological resilience (52)
		Negative self-image (32)
	Domain	Somatization (32)
	Domain	Hostility (32)
		Personality: Introversion (35)
		Personality: Low agreeableness (35)
		Personality: Openness (35)
	Domain	Stress coping: Adapted coping strategies (43)
	Domain	Hyperactivity (23)
		Internalizing behaviors (28)
		Poorer adaptive functioning (28)
Objects biting	Domain	Anxiety symptoms (44)
	Domain	Hyperactivity (23)
OBs	Domain	Anxiety symptoms (24, 27, 41, 48, 49, 57, 61–63)
		Social phobia (25)
		Stress symptoms (57, 62)
	Domain	Depression symptoms (24, 27, 48, 49, 57, 61)
	Domain	Somatosensory amplification (41, 49)
		Somatic symptoms (27)
		Somatization (62)
Sucking habits	Domain	Lower psychological resilience (52)
Toenail biting	Domain	Hyperactivity (23)

**Figure 2:**
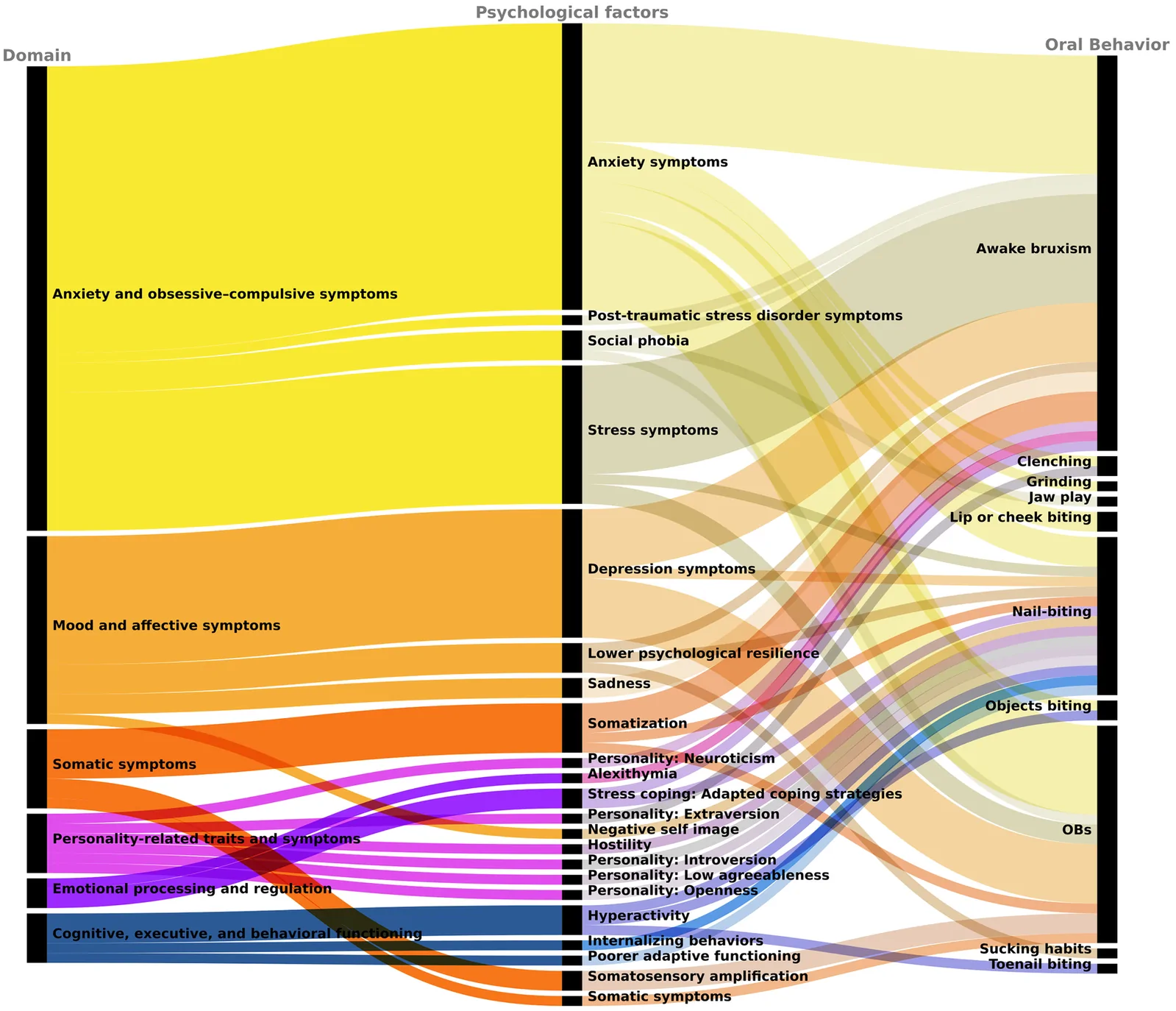
Graphical synthesis of psychological constructs organized into six analytical domains and their statistically significant associations with different oral behaviors. OBs: oral behaviors (reported as a non-divisible ensemble of diverse behaviors).

## 4 Discussion

### 4.1 Summary of evidence

This scoping review mapped the existing evidence on associations between oral behaviors and psychological factors, a topic that has long been relevant in dentistry due to its links with malocclusions (9–12), periodontal lesions (13), temporomandibular disorders (14, 63), and dental wear (64), and that may have broader implications for preventive oral healthcare and patient-centered dental practice. Although the present review was not designed to establish causality, several patterns emerged. While only a few studies reported no significant associations between oral behaviors and psychological factors, several studies identified significant associations between oral behaviors and different analytical domains. These associations varied by type of oral behavior and psychological domain, suggesting a heterogeneous and multifactorial pattern rather than a uniform relationship. In addition to their potential role in the occurrence of orofacial conditions, these findings suggest that oral behaviors may also provide clinically meaningful information regarding an individual's psychological functioning and overall well-being.

Beyond mapping isolated associations, the present review identified recurring patterns across studies that suggest oral behaviors may be better understood within an integrated biopsychological framework. The psychological constructs identified across studies were organized into six analytical domains comprising personality traits, affective symptoms (e.g., anxiety and depression), somatic symptoms, emotional regulation, and cognitive-behavioral functioning. From this perspective, oral behaviors may represent observable behavioral outputs emerging from the interaction between the psychological domains. Predisposing traits may increase vulnerability to psychological distress, while affective symptoms may act as triggers. In turn, deficits in emotional regulation or coping may facilitate the expression of repetitive oral behaviors as maladaptive strategies directed at modulating internal states. This conceptual interpretation is consistent with existing models of body-focused repetitive behaviors (1) and supports the conceptualization of oral behaviors not as isolated habits but as components of broader self-regulatory processes. Nevertheless, this conceptual interpretation should be interpreted as one plausible explanation of the observed associations rather than as evidence of causal pathways, since reverse causation, residual confounding, and shared underlying psychological determinants may also account for these relationships. To further facilitate the interpretation of these heterogeneous findings, the identified psychological constructs were organized along a distal-to-proximal continuum according to their conceptual proximity to observable oral behavior (Figure 3). Relatively stable dispositional characteristics were positioned at the more distal end of the continuum, followed by psychological states and symptoms, whereas regulatory and cognitive-behavioral processes were considered more proximal to behavioral expression. Oral behaviors constitute the observable behavioral end of the continuum. This distal-to-proximal organization is used as an analytical heuristic to distinguish constructs operating at different levels of analysis and should not be interpreted as a temporal sequence, directional pathway, or causal model. Rather, it provides an integrative structure for situating the heterogeneous associations identified across the literature. However, the available evidence does not establish such pathways, and reverse associations, shared determinants, and residual confounding remain possible.

**Figure 3:**
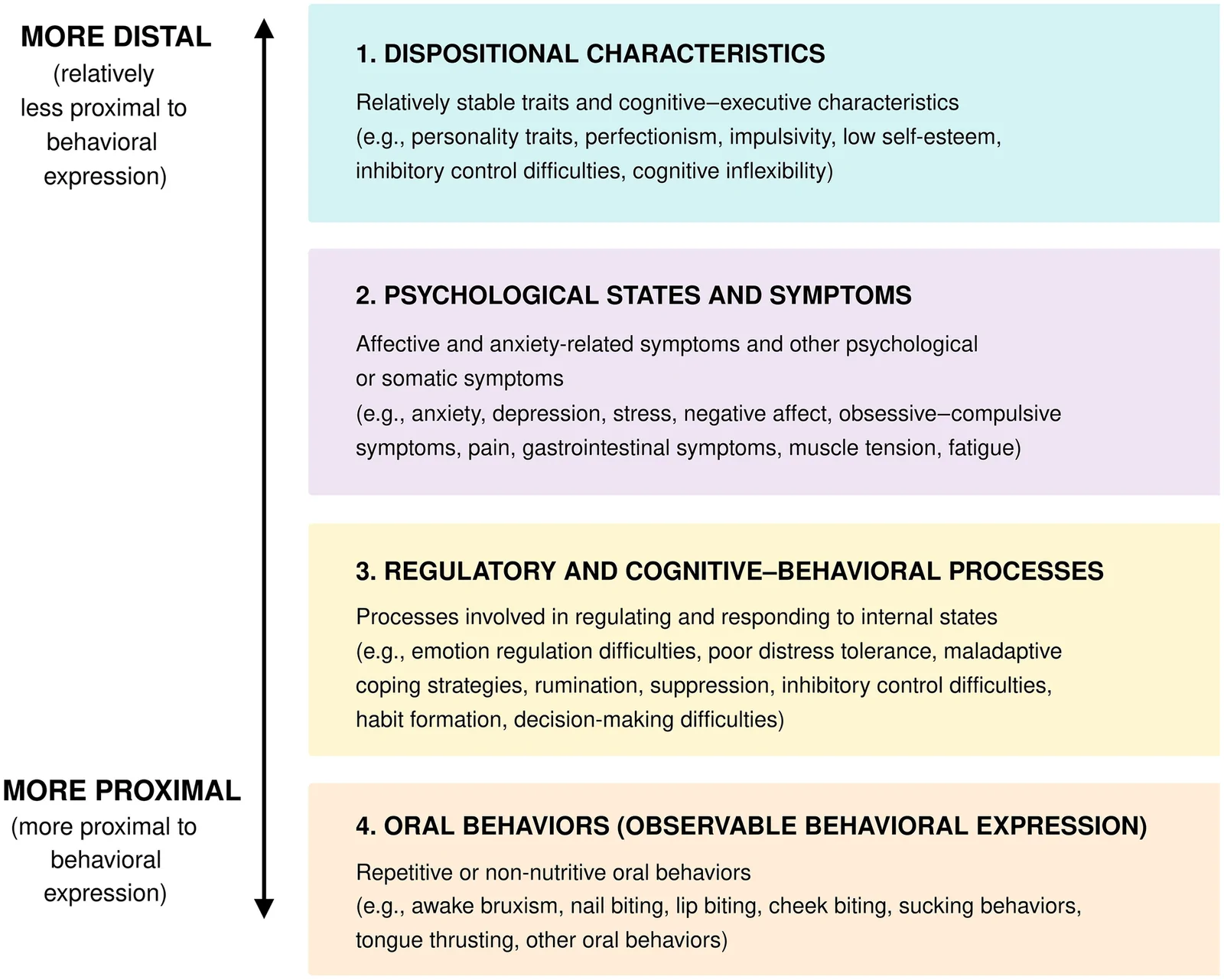
Conceptual framework of psychological constructs associated with oral behaviors organized along a distal-to-proximal continuum. Psychological constructs identified across the included studies are organized according to their conceptual proximity to observable oral behavior, ranging from relatively distal dispositional characteristics to psychological states and symptoms, regulatory and cognitive-behavioral processes, and, finally, behavioral expression. The distal-to-proximal continuum is used as an organizational heuristic and does not imply temporal ordering, directionality, or causal pathways.

### 4.2 Uneven terminology

Considerable variability was observed in the terminology used to describe oral behaviors. Although the term body-focused repetitive behaviors (BFRBs) is recognized within the DSM-5-TR taxonomy and was used in several included studies, it does not fully encompass the range of oral behaviors traditionally investigated in dental research and identified in this review which raises the question of whether oral behaviors can be considered within the BFRBs category. A recent survey of experts on BFRBs, concluded that most participant experts favor the label of body-focused repetitive behaviors as an “obsessive-compulsive and related disorder”, and also that oral behaviors, such as nail-biting and lip-cheek biting, should enter further DSM taxonomy under the category of BFRB disorders (65). Other terms—such as oral parafunctional habits, oral parafunctional behaviors, or deleterious oral habits—were often used interchangeably, with limited conceptual distinction. More recent terminology has attempted to incorporate functional and circadian aspects (e.g., non-functional waking-state oral behaviors). Given that the Oral Behavior Checklist (OBC), included in the DC/TMD Axis II, differentiates physiological from non-physiological behaviors and is widely used, the generic term oral behaviors appear to be the most pragmatic and inclusive descriptor. Importantly, the present review does not suggest that all oral behaviors should be reclassified as body-focused repetitive behaviors. Rather, the BFRB framework may offer a useful conceptual model for understanding the shared psychological mechanisms underlying several repetitive oral behaviors, particularly those related to emotional regulation, compulsivity, and repetitive self-directed actions. Nevertheless, the present findings also reinforce that oral behaviors remain a heterogeneous group of conditions with distinct diagnostic classifications, clinical characteristics, and biological mechanisms. Therefore, the BFRB framework should be interpreted as a conceptual perspective for understanding common psychological processes rather than as a proposal for a unified diagnostic classification, and the findings of this review should be interpreted as reflecting patterns across a heterogeneous group of behaviors rather than a single behavioral construct.

### 4.3 Methodological characteristics

Most of the included studies adopted cross-sectional designs and involved heterogeneous populations in terms of age (i.e., children, adolescents and/or adults), setting and clinical characteristics (i.e., students from schools and universities, general community and clinical samples that include dental, TMD, orthodontic, and psychiatric patients). This limits the ability to infer causality or estimate the strength of associations within specific subgroups. Nevertheless, the inclusion of participants across different life stages suggests that oral behaviors may occur throughout the lifespan, especially in subjects reporting high frequency of psychological impairment. Regarding the studied populations, a notable proportion of the included studies involved dental students, a group known to experience elevated levels of stress, anxiety and depression during their training period, and to possess heightened awareness of oral behaviors, which may influence self-reporting by overestimating the true extent of these behaviors (39).

### 4.4 Diagnostic aspects

The Oral Behavior Checklist (OBC) was the most frequently used instrument to assess oral behaviors. It is a self-report screening instrument for pain-relevant behavioral functioning developed by Ohrbach et al. in 2004 and is included in the DC/TMD Axis II protocol (57). Although it captures several clinically relevant physiological and non-physiological behaviors, it does not include others such as sucking habits, mouth breathing, jaw play, or toenail biting. Consequently, additional assessment instruments may be required for a comprehensive evaluation, particularly in clinical contexts not primarily involving TMD, such as malocclusions, tooth wear, or traumatic periodontal lesions. In this regard, many included studies relied on non-standardized questionnaires or insufficiently described instruments, which may introduce recall bias and limit comparability. These findings highlight the need for standardized and validated assessment tools tailored to different clinical and research contexts. This measurement heterogeneity limits direct comparisons across studies and should be considered when interpreting the variability of associations identified across the mapped evidence. Consequently, the present review focused on identifying broad patterns of evidence rather than comparing the magnitude or consistency of associations across studies.

### 4.5 Main outcomes

Awake bruxism is discussed in greater detail because it accounted for most of the available evidence identified in this review. Accordingly, interpretation of the overall findings is necessarily influenced by the predominance of studies on awake bruxism, whereas evidence regarding other oral behaviors remains considerably more limited. Awake bruxism is, by far, the most extensively investigated oral behavior in dentistry and mental health sciences. A recent systematic review reported a prevalence of 25.9% of self-reported “possible” awake bruxism and 16.0% of clinically-based “probable” awake bruxism in the general population, although substantially higher prevalence has been reported in specific subpopulations, such as subjects suffering from TMDs and certain systemic conditions (66). Awake bruxism was found to be associated with anxiety symptoms (36–38, 42, 43, 45, 50, 51, 53, 55, 56, 62), post-traumatic stress disorder symptoms (58), social phobia (25), stress symptoms (26, 29, 30, 36–38, 45, 51, 59, 60, 62), depression symptoms (42, 45, 51, 55, 56, 62), lower psychological resilience (52), sadness (30, 54), somatization (43, 45, 62), neuroticism (43), alexithymia (36), and adapted coping strategies in response to stress (43). Awake bruxism has been hypothesized to represent a transient behavioral response to biopsychological stressors and, potentially, an adaptive coping strategy through which masticatory activity may attenuate physiological and emotional stress responses (43, 62). Although experimental evidence supports stress-modulating effects of mastication, whether these mechanisms explain awake bruxism in everyday clinical settings remains uncertain. Considering the shared motor characteristics and psychological correlates of awake bruxism and other oral behaviors, it may be hypothesized that these behaviors represent similar behavioral responses to psychological or environmental challenges. However, this hypothesis remains speculative and requires further investigation.

Given the controversial results of the etiology of BFRBs, its place in the psychiatric classifications is debatable. Some authors classify these behaviors as “impulse control disorders”, while other classify these behaviors among “Obsessive Compulsive and associated disorders” although, contradictory to obsessive compulsive disorders, BFRBs are known to include emotional experiences instead of cognitive phenomenon as behavior precursors such as entrance thoughts, obsessions and mental occupation (1). Subjects engaged in BFRBs show a form of organizational perfectionism characterized by unwillingness to relax and inappropriate pacing of tasks. Individuals with this maladaptive style of planning aim to be productive at all times, setting unrealistic goals of task achievement, making them susceptible to frustration, impatience, and dissatisfaction when goals are not met (2). Subjects engaged in any BFRB (such as nail-biting) show significantly lower levels of adaptive functioning, and these behaviors may function as a maladaptive coping strategy for the internal distress (28). The emotion regulation model proposed to explain the etiopathogenesis of BFRBs (67) suggests that subjects engaged in BFRBs have difficulties in the management of unpleasant emotions, and these behaviors help to avoid, modulate, or alleviate aversive effects (2). Notably, the BFRBs may be a source of anxiety or mood-related distress, as these individuals commonly experience negative emotions such as guilt, sadness, and anger due to these behaviors (28).

Nail-biting (or onychophagia) is highly prevalent, affecting 20%–30% of the general population (13) and up to 37% of the pediatric population (68). If mild, it may not bother the patient, but when severe it may lead to physical damage and poorer quality of life (13). The relationship of nail-biting with psychological factors appears to be etiological and circumstantial. It has been postulated that nail-biting may act as an attempt to calm down in moments of stress or anxiety (22), and also as a form of self-stimulation during boredom (46). Compulsive self-injury such as nail-biting, may be an early symptom of psychiatric disease (32), in fact, nail-biters are 3.34 times more likely to have a diagnosis of a psychiatric disorder than non-biters (68). Present review found nail-biting to be significantly associated with several psychological factors such as anxiety symptoms (32, 40, 44), stress symptoms (46), depression symptoms (32), lower psychological resilience (52), negative self-image (32), somatization (32), hostility (32), certain personal traits such as introversion, low agreeableness and openness (35), adapted coping strategies to stress (43), hyperactivity (23), internalizing behaviors, and poorer adaptive functioning (28).

Some articles (24, 25, 27, 33, 41, 48, 49, 57, 61–63) did not identify specific oral behaviors in isolation but rather treated them statistically as a non-divisible ensemble of diverse behaviors, which allowed for the recognition of certain associations with psychological factors in a less precise way than other studies. This ensemble of “oral behaviors” found to be significantly associated with anxiety symptoms (24, 27, 41, 48, 49, 57, 61–63), social phobia (25), stress (57, 62), depression symptoms (24, 27, 48, 49, 57, 61), somatosensory amplification (41, 49), somatic symptoms (27), and somatization (62). The role of anxiety in oral behaviors and wake-time clenching has been largely investigated. Anxious individuals have frequent oral behaviors and wake-time clenching episodes (69). On the other hand, subjects who reported being emotionally stable are significantly less likely to have a nail-biting habit, grinding, clenching, and lip/object biting (44). Individuals with greater traits of anxiety and somatosensory amplification report more frequent oral behaviors (41). The occurrence of anxiety symptoms led to a twofold increase in the odds of having oral behaviors, while anxiety, distress and somatization were associated with a more than three times higher probability (62). Reports of oral behaviors and psychological distress have a dose-response relationship, i.e., the odds of reporting higher categories/severity of oral behaviors (e.g., clenching and grinding during waking hours, or holding the jaw in a rigid or tense position) depends on the severity of depression, anxiety, and/or stress (57).

Chow and Cioffi (2019) demonstrated a positive association between somatosensory amplification (i.e., a heightened perception of somatic sensations) and oral behaviors. Clinical studies reveal that patients with occlusal hypervigilance continuously check their occlusion. Some oral behaviors characterized by repetitive tooth-to-tooth contact, tongue-to-teeth contact, and clenching may serve to scan the intraoral environment in search of possible threats (41). Collectively, these findings suggest that oral behaviors are closely associated with psychological factors and that individuals who engage in these behaviors often present with complex psychosocial profiles, together with an increased risk of oral health problems, including malocclusions, dental wear, and temporomandibular disorders. Recent predictive models have also incorporated oral behaviors together with psychological and clinical variables to improve the identification of temporomandibular disorders (70) further reflecting the growing recognition that oral behaviors are best interpreted alongside psychological and clinical factors rather than in isolation.

### 4.6 Implications for integrated preventive oral healthcare and future research

The findings of the present review have implications that extend beyond the idea of oral behaviors as isolated parafunctional activities. The patterns of associations identified across the mapped evidence between oral behaviors and multiple analytical domains of psychological functioning suggest that these behaviors may function as clinically observable indicators of underlying psychological vulnerability. In this context, the presence of recurrent oral behaviors may prompt clinicians to consider whether psychological factors such as anxiety, mood disturbances, emotional dysregulation, or somatic symptomatology could be contributing to their occurrence. While oral behaviors are neither specific nor diagnostic of mental health disorders, their presence—particularly when frequent, severe, or associated with physical consequences—may provide valuable information regarding factors that influence oral health outcomes and overall patient well-being. Accordingly, these observations should be interpreted as hypothesis-generating rather than as evidence supporting diagnostic inferences or routine clinical decision-making.

Given that dental appointments may occur regularly over extended periods, dentists may be well positioned to recognize behavioral and psychosocial factors that remain unidentified in other healthcare settings. Routine assessment of oral behaviors, together with brief psychological screening instruments when clinically justified, may encourage clinicians to consider whether psychosocial factors could be relevant to the patient's presentation and whether further assessment is warranted. Such an approach is consistent with the growing recognition that psychological factors may influence both oral behaviors and oral health outcomes (71).

From a preventive perspective, the identification of oral behaviors may help reduce the risk of future oral health complications associated with these behaviors, including temporomandibular disorders, dental wear, traumatic periodontal lesions, and malocclusions (36, 37, 72–74). The identification of oral behaviors in patients reporting psychological distress may also provide a rationale for future preventive strategies aiming to increasing awareness of these behaviors and reducing their potential consequences (52). Although evidence regarding preventive interventions remains limited, the present findings suggest that oral behaviors may represent a clinically relevant point of contact between dentistry and mental health research (9, 32). However, whether systematic assessment of oral behaviors improves the identification of psychological distress, facilitates appropriate referral pathways, or contributes to better clinical outcomes remains to be established. Future research should determine whether systematic assessment of oral behaviors can improve the identification of psychological distress and whether this information can be meaningfully incorporated into dental assessment and management. Before such approaches can be considered for routine implementation, future studies should also evaluate their feasibility in everyday dental practice, define appropriate referral pathways, determine the training requirements for dental professionals, and validate standardized screening protocols. Addressing these implementation aspects will be essential to determine whether the observed associations can be translated into meaningful clinical practice. Longitudinal and mechanistic studies, together with the development of standardized diagnostic criteria and validated assessment instruments, are also needed to strengthen the evidence base in this field. Prospective studies should also evaluate the feasibility, diagnostic accuracy, and clinical effectiveness of integrating behavioral assessment and interdisciplinary management strategies before their routine implementation in dental practice.

### 4.7 Strengths and limitations

This scoping review presents important strengths and inherent limitations that should be considered when interpreting its findings. The review was conducted in accordance with PRISMA-ScR guidance and employed a comprehensive, reproducible search strategy across three major databases, with an updated search to capture recent evidence. Another strength is the broad geographical distribution of the included studies, enhancing the representativeness of the mapped evidence, particularly in behavioral sciences, where cultural and contextual factors may influence both psychological factors and behaviors. Furthermore, the review provides a detailed and structured mapping of a complex and heterogeneous body of literature, encompassing a wide range of oral behaviors, analytical domains, assessment methods, populations, and terminological approaches.

On the other hand, several limitations must be acknowledged. The body of evidence was mainly based on cross-sectional studies, which restricts causal inference between psychological factors and oral behaviors. Although longitudinal designs would be methodologically desirable, research in this area faces substantial practical and ethical constraints, given the difficulty of standardizing samples, the multifactorial nature of psychological exposures, and the ethical challenges associated with long-term follow-up and control conditions in behavioral research. Additional limitations include the restriction to English-language publications and to studies published within the last decade, which may have led to the omission of relevant evidence and introduced language bias. Publication bias should also be considered, as studies reporting significant associations may be more likely to be published than those with null findings. Furthermore, the heavy reliance on self-reported measures and the inclusion of heterogeneous convenience and clinical samples may have introduced reporting and selection biases, respectively. Although the review protocol was developed before study selection, it was not prospectively registered or made publicly available. Consequently, the transparency of the review process may have been reduced, and *post hoc* modifications to the review methods cannot be independently excluded. These methodological characteristics, together with the potential influence of residual confounding, the potential selection bias arising from the inclusion of heterogeneous study populations, and differences in measurement validity across studies, should also be considered when interpreting the consistency of the reported associations, as the number of studies reporting a particular finding does not necessarily reflect the strength, reliability, or certainty of the available evidence. Finally, as is inherent to scoping review methodology, no assessment of methodological quality or risk of bias was performed, and the findings should therefore be interpreted as an exploratory mapping of the literature rather than an evaluation of effect magnitude or certainty of evidence. Overall, the available evidence shows recurrent reports of associations between oral behaviors and psychological factors across a broad range of studies. Most included studies reported statistically significant associations involving multiple psychological constructs and different oral behaviors, suggesting that these relationships are not restricted to a single psychological dimension or behavioral phenotype. However, the evidence base remains methodologically heterogeneous, is dominated by cross-sectional observational studies using diverse terminology and assessment methods and therefore represents an emerging rather than mature body of evidence. Consequently, the certainty with which these associations can be interpreted remains limited, and further longitudinal studies using standardized terminology, assessment methods, and reporting approaches are needed before more definitive conclusions can be drawn.

## 5 Conclusion

Current evidence suggests that oral behaviors are associated with psychological factors spanning multiple analytical domains, particularly “anxiety and obsessive–compulsive symptoms” and “mood and affective symptoms”. Rather than isolated “bad habits”, these behaviors may occur within broader biopsychosocial patterns that are relevant for both dental and mental health. However, the evidence base remains predominantly cross-sectional and methodologically heterogeneous, limiting causal inference and the certainty with which these associations can be interpreted. These findings suggest that oral behaviors may warrant consideration alongside psychological functioning and support further research toward more integrated and interdisciplinary approaches.

## Data Availability

The original contributions presented in the study are included in the article/Supplementary Material, further inquiries can be directed to the corresponding author.
