# Supplementary material for: Mapping the evidence on oral behaviors and mental health: a scoping review with implications for integrated oral healthcare
Source: Front Oral Health. 2026 Oct 1;7:1906938. doi: 10.3389/froh.2026.1906938 (PMC13668535; doi:10.3389/froh.2026.1906938)
Supplement: Supplementary file 1 [file Datasheet1.pdf]

Reference	DOI	Country	Study design	Total No. of included subjects	Included subjects phase of life	Included subjects age range (in years)	Included subjects age mean (in years)	Subjects of study descriptive characteristic	Terminology used to refer to "Oral behaviors"	Oral behavior assessed	Oral behavior assessment method	Psychological feature assessed (method of assessment)	Other non-oral Body-focused repetitive behaviors (BFRBs) assessed	Predictive multivariate model with OB as main outcome (Y/N)	Statistically significant associations of OBs and psycological features	Significant associations of BFRBs (involving OB) and psychological feature
Pacan et al., 2014	10.2340/00015555-1616	Poland	Cross-sectional	339	Adults	21-26	23.9 ± 1.1	University students	NR	NB	•Questionnaire	•Anxiety and obsesive compulsive disorder symptoms (Composite International Diagnostic Interview, CIDI)	Onychotillomania	N	*No significant associations for psychological factors*	NR
Sabuncuoglu et al., 2014	10.1089/bfm.2014.0014	Turkey	Case-control	375	Children and adolescents	7-17	10.25 ± 2.52	ADHD patients and healthy controls	Parafunctional Oral Habits	Finger sucking, NB, toenail biting, Pencil biting, Cheek biting, mouth breathing	•Questionnaire (Y/N questions and frequencies)	•Hyperactivity, inattentiveness, oppositional behavior, psychosomatic behavior, and irritability (Conners Parent Rating Scale, CPRS)<br>•Hyperactivity, inattentiveness, and conduct problems (Conners Teacher Rating Form, CTRF)	N	N	•NB: Hyperactivity<br>•Pencil biting: Hyperactivity<br>•Toenail biting: Hyperactivity	NR
Leme et al., 2014	10.17796/jcpd.38.4.c48238322205466w	Brazil	Cross-sectional	147	Children and adolescents	8-14	NR	School students	Deleterious Oral Habits	OBs	•Nordic Orofacial Test-Screening (NOT-S)	•Anxiety symptoms (Revised Children's Manifest Anxiety Scale, RCMAS)<br>•Depression symptoms (Children's Depression Inventory, CDI)	N	Y	•OBs: anxiety symptoms, and symptoms of depression	NR
Hermesh et al., 2015	10.1111/joor.12235	Israel	Cross-sectional	73	Adults	20-65	NR	Social phobia subjects and healthy controls	Oral Parafunctional hyperactivity	AB, OBs, and jaw play	•Questionnaire (Y/N questions)	•Social Phobia (The Liebowitz Social Phobia Symptom Scale, LSAS)<br>•Anxiety symptoms (The Agoraphobic Cognition Questionnaire, ACQ)<br>•Anxiety symptoms (Body Sensations Questionnaire, BSQ)<br>•Depression symptoms (The Beck Depression Inventory, BDI)	N	Y	•AB: social phobia<br>•Jaw play: social phobia<br>•OBs: social phobia	NR
Quadri et al., 2015	10.1155/2015/842096	Saudi Arabia	Cross-sectional	476	Adolescents and adults	NR	NR	College students	NR	AB	•Questionnaire (Y/N questions)	•Stress symptoms (Questionnaire)	N	Y	•AB: stress	NR
Khawaja et al., 2015	10.1111/joor.12302	USA	Cross-sectional	94	Adults	NR	34.2 ± 12.1	General community	Oral parafunctional behaviors	OBs	•Oral behavioral Checklist (OBC) from DC/TMD	•Anxiety symptoms (General anxiety disorder-7, GAD-7)<br>•Depression symptoms (Physical health questionnaire-9, PHQ-9)<br>•Somatic symptoms (Physical health questionnaire-15, PHQ-15)	N	Y	•OBs: anxiety symptoms, depression symptoms, and somatic symptoms	NR
Selles et al., 2015	10.1016/j.jocrd.2015.01.008	El Salvador	Cross-sectional	315	Children and adolescents	4-17	8.39 ± 3.10	General community	Body-focused repetitive behaviors	NB	•Repetitive Body Focused Behavior Scale - Parents (RBFBS)	•Behavioral and Emotional Functioning (Behavioral Assessment System for Children – II: Behavioral and Emotional Screening System – Parent Form – Child/Adolescent, BESS)	Hair-pulling, skin-picking	N	•NB: internalizing behaviors, and poorer adaptive functioning	NR
Cavallo et al., 2016	10.1186/s13104-016-2311-0	Italy	Cross-sectional	278	Adults	NR	23,7	University students	NR	AB	•Questionnaire (Likert-type scale)	•Stress symptoms (Perceived Stress Scale, PSS-10)	N	N	•AB: stress	NR
Emodi Perlman et al., 2016	10.1111/joor.12391	Israel	Cross-sectional	1.000	Adolescents	12–18	15.1 ± 1.5	High school students	NR	AB	•Questionnaire (Y/N questions)	•Stress symptoms (Questionnaire Y/N questions)<br>•Sadness symptoms (Questionnaire Y/N questions)	N	Y	•AB: "feeling stressed", and "feeling sad"	NR
Blum et al., 2017	10.1007/s11126-017-9496-9	USA	Case-Control	87	Adults	18-29	NR	General community	Body-focused repetitive behaviors	NB	•Questionnaire	•Depression symptoms (Hamilton Depression Rating Scale, HAM-D)<br>•Anxiety symptoms (Hamilton Anxiety Rating Scale, HAM-A)<br>•Cognitive flexibility (Intra-Extra Dimensional Set Shift, IDED)<br>•Response inhibition (Stop-Signal Task, SST)	N	N	*No significant associations for psychological factors*	NR
Sisman et al., 2017	10.1177/0143034317690578	Turkey	Cross-sectional	724	Adolescents	11-17	NR	Secondary and high schools students	Body-focused repetitive behaviors	NB	•Questionnaire (Y/N questions)	•Anxiety, depression, negative self, somatization, hostility, Global Severity Index, Positive Symptom Distress Index, and Positive Symptom Total (Brief Symptom Inventory, BSI)	N	N	•NB: anxiety, depression, negative self image, somatization, and hostility.	NR
Çoban Buyukbayraktar and Doruk, 2017	10.7126/cumudj.341904	Turkey	Cross-sectional	135	Children and adolescents	9-12	NR	Subjects applying to orthodontic clinic	Deleterious Oral Habits	OBs	•Questionnaire and clinical examination	•Depression symptoms (Children's Depression Inventory, CDI)<br>•Anxiety symptoms (Children's State Trait Anxiety Inventory, CSTAI)	N	N	*No significant associations for psychological factors*	NR
Solley and Turner, 2018	10.1016/j.comppsych.2018.06.014	Australia	Cross-sectional	1.378	Adults	18-91	28.88 ± 11.66	University students	Body-focused repetitive behaviors	NB, lip or cheek biting	•Massachusetts General Hospital Hair-Pulling Scale (MGH- HPS) analogue for nail biting, cheek biting and chewing behaviors.	•Depression symptoms (Patient Health Questionnaire, PHQ-9)<br>•Physiological experience of anxiety (Anxiety subscale from Depression Anxiety Stress Scale-21 , DASS-A)<br>•Anxiety (Generalized Anxiety Disorder-7, GAD-7)<br>•Obsessive-compulsive symptoms (Obsessive Compulsive Inventory-Revised, OCI-R)<br>•Symptoms of social phobia and anxious feelings in relation to social interactions (Social Interaction Anxiety Scale, SIAS-6; and Social Phobia Scale, SPS-6)<br>•Fear of negative evaluation (Brief fear of negative evaluation scale, bFNE)<br>•Dysmorphic concern (Body Image Concerns Inventory, BICI)	Hair-pulling, skin-picking	N	*No significant associations for psychological factors*	•BFRBs: anxiety, depression, Obsessive-compulsive symptoms, body dysmorphic disorder, and fear of negative evaluation.
Oshio, 2018	10.1007/s12646-018-0462-x	Japan	Cross-sectional	5.328	Adults	18-71	49.9 ± 11.0	General community	Body-focused repetitive behaviors	NB	•Questionnaire (Likert-type scale)	•Personality traits (Ten-Item Personality Inventory, TIPI-J)	Leg-shaking	Y	•NB: Introversion, low agreeableness and openness	NR
Przystanska et al., 2019	10.1155/2019/2069716	Poland	Cross-sectional	52	NR	NR	NR	TMD patients	NR	AB	•Questionnaire (Y/N questions) and clinical assessment	•Alexithymia (Toronto Alexithymia Scale, TAS- 20)<br>•Emotional processing (Emotional Processing Scale, EPS)<br>•Stress (Perceived Stress Scale, PSS-10)<br>•Anxiety (State- and Trait-Anxiety Inventory, STAI)	N	N	•AB: state anxiety, trait anxiety, stress, emotional processing deficits, and alexithymia	NR
Winocur et al., 2019	10.3389/fneur.2019.00443	Israel	Cross-sectional	2.347	Adolescents	14-18	15.7 ± 1.1	High school students	NR	AB	•Questionnaire (Y/N questions)	•Anxiety symptoms (General anxiety disorder-7, GAD-7)<br>•Stress symptoms (Perceived Stress Scale-10, PSS-10)	N	Y	•AB: anxiety, and distress	NR
Gouw et al., 2019	10.1111/joor.12727	Netherlands	Longitudinal observational study	55	Adults	NR	28.4 ± 12.1	General community	NR	AB	•Diary with Numeric Rating Scales (NRS) for the experienced level of awake bruxism	•Stress symptoms (Perceived Stress Scale, PSS)<br>•Anxiety symptoms (State Trait Anxiety Inventory, STAI)<br>•Anger symptoms (State Trait Anger Expression Inventory, STAXI)	N	Y	•AB: anxiety, and distress	NR
Jokubauskas et al., 2019	10.1080/07420528.2019.1660356	Lithuania	Cross-sectional	228	Adults	NR	22.67 ± 2.27	Dentistry students	NR	AB	•Questionnaire (Y/N questions)	•Stress symptoms (Perceived Stress Scale-10, PSS-10)	N	Y	*No significant associations for psychological factors*	NR
Murphy et al., 2019	10.1016/j.jocrd.2018.12.002	USA	Cross-sectional	530	Adults	NR	37.80 ± 7.93	Parents/caregivers of youths	Body-focused repetitive behaviors	NB, lip-biting	•Child Health and Anxiety Survey (CHAS)	•Anxiogenic parenting (The Parenting Anxious Kids Ratings Scale - Parent Report, PAKRS-PR)<br>•Child anxiety (Spence Children's Anxiety Scale, SCAS)<br>•Depression, anxiety and stress (Depression Anxiety Stress Scale, DASS-21)	Hair-pulling, skin-picking	Y	•NB: child anxiety, anxiogenic parenting behaviors (accommodation and negative beliefs).<br>•LB: child anxiety, anxiogenic parenting behaviors	•BFRBs: child anxiety, anxiogenic parenting behaviors
Chow & Cioffi, 2019	10.1007/s00784-018-2600-1	Canada	Cross-sectional	255	Adults	NR	NR	University students	Oral behaviors	OBs	•Oral Behavior Checklist (OBC)	•Anxiety symptoms (State and Trait Anxiety Inventory, STAI)•Somatic amplification (Somatosens	N	Y	•OBs: somatosensory amplification, and anxiety	NR
Machado et al., 2020	10.1590/1678-2019-0407	Brazil	Prospective Cohort	114	Adolescents and adults	13-60	24,7	Subjects using fixed orthodontics appliance	Oral parafunctional behaviors	AB	•Oral Behavioral Checklist (OBC) from DC/TMD	•Attention to pain (Pain vigilance and awareness questionnaire, PVAQ).<br>•Severity of anxiety symptoms (Beck anxiety inventory, BAI)<br>•Severity of depressive symptoms (Beck depression inventory, BDI)	N	N	•AB: anxiety, and depression	NR
Soto-Goñi et al., 2020	10.3389/fneur.2020.564431	Spain	Case-control	68	Adolescents and adults	17-31	19,6	University students	NR	AB	•Questionnaire (Y/N questions)	•Anxiety (State and Trait Anxiety Inventory, STAI)<br>•Depression (State and Trait Depression Inventory, ST-DEP)<br>•Stress coping (Coping Response Inventory—Adult form, CRI-A)<br>•Symptoms of depression, anxiety and somatization (Brief Symptom Inventory: Anxiety, Depression and Somatization, BSI-18)<br>•Personality traits (NEO Personality Inventory, NEO-FFI)	N	N	•AB: anxiety (state, trait, and symptoms), somatization, neuroticism, adapted coping strategies	NR
Almutairi et al., 2021	10.1016/j.sdentj.2020.01.003	Saudi Arabia	Cross-sectional	1.263	Adults	NR	NR	General community	Oral parafunctional habits	NB, grinding, clenching, lip/objects biting, and gum chewing	•Questionnaire (frequency scale)	•Anxiety symptoms (Hamilton Anxiety Rating Scale, HAM-A)<br>•Big-Five Personality factors (Ten-Item Personality Inventory)	N	Y	•NB: anxiety<br>•Grinding: anxiety<br>•Clenching: anxiety, extraversion personality<br>•Lip/object biting: anxiety	NR
Levartovsky et al., 2021	10.3390/ jcm11010010	Israel	Cross-sectional	387	Adults	NR	24 ± 2	Dentistry students	NR	AB	•Questionnaire (Y/N questions)	•Stress (Perceived Stress Scale Questionnaire 14, PSS14)<br>•Psychological and psychiatric symptoms (Symptom Checklist-90, SCL-90)	N	N	•AB: somatization, depression, anxiety, and stress	NR
Erdogan et al., 2021	10.15570/actaapa.2021.4	Turkey	Cross-sectional	3.475	Adolescents and adults	14-40	NR	High school and university students	Body-focused repetitive behaviors	NB	•Questionnaire	•Stress (Perceived Stress Scale, PSS)	N	N	•NB: stress	NR
Houazene et al., 2021	10.1017/bec.2020.16	Canada	Cross-sectional	76	Adults	18-65	35	General community	Body-focused repetitive behaviors	NB	•Massachusetts General Hospital Hair-Pulling Scale (MGH- HPS) analogue for nail-biting	•Perfectionism (Frost Multidimensional Perfectionism Scale, FMPS)<br>•Dependency and self-criticism (Depressive Experiences Questionnaire, DEQ)<br>•Shame (Experience of Shame Scale, ESS)<br>•Cognitive emotion regulation strategies (Cognitive Emotion Regulation Questionnaire, CERQ)<br>•Emotion regulation difficulties (Difficulties in Emotion Regulation Scale, DERS)	Hair-pulling, skin-picking	Y	•NB: maladaptive cognitive emotion regulation strategies	•BFRBs: maladaptive strategies, shame
Xu et al., 2021	10.12659/MSM.929985	China	Cross-sectional	537	Adolescents and adults	15-68	31.55 ± 12.08	TMD patients	Oral behaviors	OBs	•Oral Behavior Checklist (OBC)	•Depression symptoms (Patient Health Questionnaire, PHQ-9)<br>•Anxiety symptoms (General Anxiety Disorder Scale-7, GAD-7)	N	N	•OBs: anxiety, and depression	NR
Vrbanovic et al., 2022	10.1080/00016357.2022.2042380	Croatia	Cross-sectional	100	Adults	NR	NR	Dental patients	Oral parafunctional behaviors	OBs	•Oral Behavior Checklist (OBC)	•Depression symptoms (Patient Health Questionnaire, PHQ-9)<br>•Anxiety symptoms (General Anxiety Disorder Scale-7, GAD-7)<br>•Somatic amplification (Somatosensory Amplification Scale, SSAS)	N	Y	•OBs: somatosensory amplification, depression symptoms, and anxiety symptoms	NR
de Oliveira et al., 2023	10.4103/jid.jid_23_22	Brazil	Cross-sectional	18	Adults	19-46	32	General community	NR	AB	•Oral Behavior Checklist (OBC)<br>•Ecological momentary assessment (SKEDit app)	•Self-assessed Stress (Perceived Stress Scale, PSS)<br>•Anxiety (Generalized Anxiety Disorder Scale, GAD-7)	N	N	•AB: anxiety	NR
Câmara-Souza et al., 2023	10.1080/08869634.2020.1829289	Brazil	Cross-sectional	69	Adults	NR	18.6 ± 1.5	College students	NR	AB	•Oral Behavior Checklist (OBC)	•Anxiety and depression (Hospital Anxiety and Depression Scale, HADS)<br>•Stress (Perceived Stress Scale, PSS)	N	N	•AB: anxiety, depression, and stress	NR
Abd-Elsabour et al., 2023	10.1038/s41415-023-5565-7	Egypt	Cross-sectional	227	Children	5-7	NR	School students	Oral habits	NB, AB, and SHab	•Nordic Orofacial Test-Screen (NOT-S)<br>•Bruxapp	•Child's psychological resilience (Child and Youth Resilience Measure-Revised Person Most Knowledgeable version, PMK-CYRM-R)	N	N	•AB: lower personal resilience<br>•NB: lower personal resilience<br>•SHab: lower personal resilience	NR
Hilgenberg-Sydney et al., 2024	10.1055/s-0044-1782179	Brazil	Cross-sectional	104	Adults	18-35	22,34	Dentistry students	Oral parafunctional habits	AB	•Oral Behavior Checklist (OBC)	•Anxiety (State-Trait Anxiety Inventory, STAI)	N	Y	•AB: anxiety	NR
Figueiroa et al., 2024	10.1590/pboci.2024.081	Brazil	Cross-sectional	1.603	Adolescents	14-19	NR	School students	NR	AB	•Research Diagnostic Criteria for Temporomandibular Dysfunction (RDC/TMD) questionnaire	•Sadness (Youth Risk Behavior Survey, YRBS)	N	Y	•AB: feeling of sadness	NR
Saracutu et al., 2024	10.1080/08869634.2024.2357199	Italy	Cross-sectional	100	Adults	19–29	22.5 ± 2.5	Dentistry students	NR	AB	•Bruxapp	•Anxiety and depression (Patient Health Questionnaire-4, PHQ-4)	N	N	•AB: anxiety and depression	NR
Saracutu et al., 2024 (2)	10.22514/jofph.2024.041	Italy	Cross-sectional	100	Adults	19–29	22.5 ± 2.5	Dentistry students	NR	AB	•Oral Behavior Checklist (OBC)	•Anxiety and depression (Patient Health Questionnaire-4, PHQ-4)	N	N	•AB: anxiety and depression	NR
van Selms & Lobbezoo, 2024	10.1080/08869634.2024.2360865	Netherlands	Retrospective Cross-sectional	1.886	Adults	NR	42.4 ±15.3	TMD patients	Non-functional waking-state oral behaviors	OBs	•Oral Behavior Checklist (OBC)	•Depression (Patient Health Questionnaire, PHQ-9)<br>•Anxiety (Generalized Anxiety Disorder-7, GAD-7)<br>•Stress (Questionnaire)	N	Y	•OBs: depression, anxiety, and stress	NR
Solis et al., 2024	10.1007/s00784-024-05534-4	Brazil	Case-control	76	Adults	NR	NR	PTSD patients and control subjects	NR	AB	•Questionnaire	•Post-traumatic stress disorder (Structured Clinical Interview for DSM-IV-TR Axis I Disorder, SCID-I)<br>•Post-traumatic stress disorder symptoms (Davidson Trauma Scale, DTS)<br>•Depression (Symptom Checklist 90-R, SCL-90-R)	N	Y	•AB: Post-traumatic stress disorder	NR
Fluerasu et al., 2024	10.62610/RJOR.2024.4.16.17	Romania	Cross-sectional	271	Adults	18-65	NR	General community	NR	AB	•Questionnaire	•Depression, Anxiety and Stress (Depression Anxiety Stress Scale-21, DASS-21)	N	Y	•AB: stress	NR
Souza & Cavalcanti, 2024	10.1590/pboci.2024.059	Brazil	Cross-sectional	391	Adults	NR	NR	Dentistry students	NR	AB	•Questionnaire (Y/N questions)	•Stress (Perceived Stress Scale, PSS-10)	N	Y	•AB: stress	NR
Yap et al., 2024	10.1007/s00784-024-05730-2	South Korea	Cross-sectional	700	Adults	NR	37.4 ± 15.7	TMD patients	Oral behaviors	OBs	•Oral Behavior Checklist (OBC)	•Depression (Patient Health Questionnaire, PHQ-9)<br>•Anxiety (General Anxiety Disorder Scale-7, GAD-7)	N	Y	•OBs: anxiety, and depression	NR
Restrepo-Serna et al., 2024	10.1080/08869634.2024.2441526	Colombia	Cross-sectional	79	Adolescents	12-15	13.5 ± 1.6	Pediatric Dentistry and Orthodontics patients	Non-physiological awake oral behaviors	OBs, AB	•Oral Behavior Checklist (OBC)	•Symptoms of depression (Patient Health Questionnaire for Adolescents, PHQ-A)<br>•Symptoms of anxiety (Generalized Anxiety Disorder-7, GAD-7)<br>•Distress (Perceived Stress Scale-Children, PSS-C)<br>•Somatization (Patient Health Questionnaire, PHQ-15)	N	Y	•AB: anxiety, distress, somatization, depression<br>•OBs: anxiety, distress, and somatization	NR
Michelotti et al., 2025	10.1016/j.archoralbio.2025.106321	Italy	Retrospective Cross-sectional	743	Adults	NR	NR	TMD patients and healthy controls	Non-functional oral behaviors	OBs	•Oral Behavior Checklist (OBC)	•Symptoms of anxiety (Generalized Anxiety Disorder-7, GAD-7)	N	Y	•OBs: anxiety	NR
