# Supplementary material for: Mapping the evidence on oral behaviors and mental health: a scoping review with implications for integrated oral healthcare
Source: Front Oral Health. 2026 Oct 1;7:1906938. doi: 10.3389/froh.2026.1906938 (PMC13668535; doi:10.3389/froh.2026.1906938)
Supplement: Supplementary file 2 [file Table1.docx]

TABLE. Search strategies tailored for each database.

| **Database** | **Search string** |
| --- | --- |
| Pubmed | ((((((((((((((((((((((((("bad oral habit"[tiab:~0]) OR ("oral parafunction")) OR ("parafunctional oral habit")) OR ("oral parafunctional behavior"[tiab:~0])) OR ("dysfunctional oral habit"[tiab:~0])) OR ("oral parafunctional activity")) OR ("harmful oral habits")) OR ("deleterious oral habit")) OR ("abnormal oral habit"[tiab:~0])) OR ("compulsive oral habit"[tiab:~0])) OR ("Masochistic habits")) OR ((((("nail biting") OR ("onychophagia")) OR ("nailbiting")) OR ("nail tic disorders")) OR ("pathological nail biting"))) OR (("lip biting") OR ("lip-biting"))) OR (("cheeks biting"[tiab:~0]) OR ("cheek chewing"))) OR (("pencil biting") OR ("object biting"))) OR ("tongue biting")) OR (((("deleterious sucking habits") OR ("non-nutritive sucking")) OR ("non-nutritive sucking habits")) OR ("non-nutritive sucking behaviors"))) OR ((("thumb sucking") OR ("fingersucking")) OR ("finger sucking"))) OR (("pacifier sucking") OR ("pacifier habit"))) OR ((("lower lip sucking habit") OR ("lip sucking")) OR ("lip licking"))) OR ((((("tongue thrusting") OR ("tongue thrusting habit")) OR ("tongue-thrusting")) OR ("tongue-thrusting habit")) OR ("tongue thrust"))) OR (((((("bruxism") OR ("awake bruxism")) OR ("daytime bruxism")) OR ("teeth clenching")) OR ("teeth grinding")) OR ("clenching"))) NOT ("oral hygiene habits")) NOT ("oral hygiene behavior")) NOT ("tooth brushing habits")) NOT ("dental caries") AND (2014:2025[pdat]) Filters: English |
| Web of science | (((((((((((((((((((((((((((((((((((((((((ALL=("bad oral habit")) OR ALL=("oral parafunction")) OR ALL=("parafunctional oral habit")) OR ALL=("oral parafunctional behavior")) OR ALL=("dysfunctional oral habit")) OR ALL=("oral parafunctional activity")) OR ALL=("harmful oral habits")) OR ALL=("deleterious oral habit")) OR ALL=("abnormal oral habit")) OR ALL=("compulsive oral habit")) OR ALL=("nail biting")) OR ALL=("onychophagia"))) OR ALL=("nailbiting")) OR ALL=("nail tic disorders")) OR ALL=("pathological nail biting")) OR ALL=("lip biting")) OR ALL=("lip-biting")) OR ALL=("cheeks biting")) OR ALL=("cheek chewing")) OR ALL=("tongue biting")) OR ALL=("pencil biting")) OR ALL=("object biting")) OR ALL=("deleterious sucking habits")) OR ALL=("non-nutritive sucking")) OR ALL=("non-nutritive sucking habits")) OR ALL=("non-nutritive sucking behaviors")) OR ALL=("thumb sucking")) OR ALL=("fingersucking")) OR ALL=("finger sucking")) OR ALL=("pacifier sucking")) OR ALL=("pacifier habit")) OR ALL=("lower lip sucking habit")) OR ALL=("lip sucking")) OR ALL=("lip licking")) OR ALL=("bruxism")) OR ALL=("awake bruxism")) OR ALL=("daytime bruxism")) OR ALL=("teeth clenching")) OR ALL=("teeth grinding")) OR ALL=("clenching")) OR ALL=("Masochistic habits") |
| Scopus | ( TITLE-ABS-KEY ( "bad oral habit" OR "dysfunctional oral habit" OR "harmful oral habits" OR "deleterious oral habit" OR "abnormal oral habit" OR "compulsive oral habit" OR "oral parafunction" OR "parafunctional oral habit" OR "oral parafunctional behavior" OR "parafuntional activity" OR "masochistic habits" OR "nail biting" OR "onychophagia" OR "nailbiting" OR "nail tic disorders" OR "pathological nail biting" OR "lip biting" OR "lip-biting" OR "cheeks biting" OR "cheek chewing" OR "pencil biting" OR "object biting" OR "tongue biting" OR "deleterious sucking habits" OR "non-nutritive sucking" OR "non-nutritive sucking habits" OR "non-nutritive sucking behaviours" OR "thumb sucking" OR "finger sucking" OR "pacifier sucking" OR "pacifier habit" OR "lower lip sucking" OR "lip sucking" OR "lip licking" OR "tongue thrusting" OR "tongue thrusting habit" OR "tongue-thrusting" OR "tongue-thrusting habit" OR "tongue thrust" OR "bruxism" OR "awake bruxism" OR "daytime bruxism" OR "teeth clenching" OR "clenching" ) AND PUBYEAR > 2013 AND PUBYEAR < 2026 ) AND NOT ( ALL ( "oral hygiene habits" ) ) AND NOT ( ALL ( "oral hygiene behavior" ) ) AND NOT ( ALL ( "tooth brushing habits" ) ) AND NOT ( ALL ( "dental caries" ) ) AND PUBYEAR > 2013 AND PUBYEAR < 2026 AND ( LIMIT-TO ( LANGUAGE , "english" ) OR LIMIT-TO ( LANGUAGE , "spanish" ) ) AND ( EXCLUDE ( subjarea , "envi" ) OR EXCLUDE ( subjarea , "arts" ) OR EXCLUDE ( subjarea , "phys" ) OR EXCLUDE ( subjarea , "busi" ) OR EXCLUDE ( subjarea , "econ" ) OR EXCLUDE ( subjarea , "math" ) OR EXCLUDE ( subjarea , "vete" ) OR EXCLUDE ( subjarea , "comp" ) OR EXCLUDE ( subjarea , "engi" ) OR EXCLUDE ( subjarea , "ener" ) OR EXCLUDE ( subjarea , "deci" ) OR EXCLUDE ( subjarea , "agri" ) OR EXCLUDE ( subjarea , "ceng" ) OR EXCLUDE ( subjarea , "mate" ) ) |
