# Supplementary material for: Mapping the evidence on oral behaviors and mental health: a scoping review with implications for integrated oral healthcare
Source: Front Oral Health. 2026 Oct 1;7:1906938. doi: 10.3389/froh.2026.1906938 (PMC13668535; doi:10.3389/froh.2026.1906938)
Supplement: Supplementary file 3 [file Table2.docx]

Supplementary Table. Operational definitions and categorization of key data items used in the scoping review.

| **Data item** | **Operational definition or categorization** |
| --- | --- |
| Oral behaviors | Repetitive or non-nutritive oral activities explicitly assessed in the included studies. |
| Individual oral behaviors | Behaviors were categorized according to the terminology reported in the original studies; clearly equivalent terms were harmonized for evidence mapping. The principal harmonized categories were awake bruxism, nail-biting, lip/cheek biting, sucking habits, object biting, jaw play, and non-divisible ensembles reported generically as oral behaviors. |
| Psychological factors | Psychological, affective, cognitive, personality-related, somatic, and regulatory constructs assessed in relation to oral behaviors. |
| Analytical domains | Six review-specific categories based on conceptual similarity: *Anxiety and obsessive–compulsive symptoms; Mood and affective symptoms; Somatic symptoms; Personality-related traits and symptoms; Emotional processing and regulation; Cognitive, executive, and behavioral functioning*. |
| Psychological assessment method | **Validated psychometric instrument:** standardized instrument with previously established psychometric properties reported or referenced by the original study.  **Diagnostic assessment:** assessment based on established diagnostic criteria or a structured/clinical diagnostic procedure.  **Investigator-developed questionnaire:** questionnaire developed or adapted specifically for the study without established validation reported.  **Single-item/non-standardized self-report measure:** direct self-reported assessment not constituting a standardized psychometric instrument. |
| Association | Statistically significant relationship reported by the original study between an oral behavior and a psychological factor, including correlation, between-group difference, or regression-based association. |
